# Social cognition in myotonic dystrophy type 1: Specific or secondary impairment?

**DOI:** 10.1371/journal.pone.0204227

**Published:** 2018-09-24

**Authors:** Garazi Labayru, Irati Arenzana, Jone Aliri, Miren Zulaica, Adolfo López de Munain, Andone Sistiaga A.

**Affiliations:** 1 Neuroscience Area, Biodonostia Research Institute, Donostia-San Sebastian, Gipuzkoa, Spain; 2 Personality, Assessment and Psychological Treatment Department, Psychology Faculty, University of the Basque Country (UPV/EHU), Donostia-San Sebastian, Gipuzkoa, Spain; 3 Master’s in Child and Adult Neuropsychology, Autonoma University of Barcelona, Spain; 4 Department of Social Psychology and Methodology of Behavioral Sciences, University of the Basque Country (UPV/EHU), Donostia-San Sebastian, Gipuzkoa, Spain; 5 Neurology Department, Donostia University Hospital, Donostia-San Sebastian, Gipuzkoa, Spain; Heriot-Watt University School of Social Sciences, UNITED KINGDOM

## Abstract

**Aims:**

The cognitive profile of Myotonic Dystrophy type 1 (DM1) has been described in recent decades. Moreover, DM1 patients show lowered social engagement and difficulties in social-cognitive functions. The aim of the present study is to explore whether social cognition impairment is present in DM1 taking into account the overall cognitive condition.

**Method:**

38 patients and a control group paired in age and gender participated in the study. All the participants had an IQ within the normal range. Subjects were administered an abbreviated neuropsychological battery which comprised a facial emotion recognition test (POFA) and Faux Pas Test, as well as a self-report questionnaire on cognitive and affective empathy (TECA).

**Results:**

Statistically significant differences were found only for facial emotion recognition (*U* = 464.0, *p* = .006) with a moderate effect size (.31), with the controls obtaining a higher score than the patients. Analyzing each emotion separately, DM1 patients scored significantly lower than controls on the recognition of anger and disgust items. Emotion recognition did not correlate with genetic load, but did correlate negatively with age. No differences were found between patients and controls in any of the other variables related to Theory of Mind (ToM) and empathy.

**Conclusion:**

DM1 does not manifest specific impairments in ToM since difficulties in this area predominantly rely on the cognitive demand of the tasks employed. However, a more basic process such as emotion recognition appears as a core deficit. The role of this deficit as a marker of aging related decline is discussed.

## Introduction

Myotonic dystrophy type 1 (DM1) is a slowly progressive muscular dystrophy characterized by multisystemic involvement [1]. As the most prevalent neuromuscular disorder, it shows a particularly high-prevalence focus in Gipuzkoa (North of Spain), reaching 300 cases per million inhabitants [2].

Previous genotype–phenotype correlation studies in DM1 have reported an association between the CTG repeat size (the molecular defect characterizing DM1) and certain clinical features, such as cognitive impairment and muscular weakness, as well as the age of onset [3–5]. In this regard, there are data supporting the possibility that the expansion size correlates with the cognitive impairment, particularly if the whole DM1 spectrum is taken into account, which ranges from intellectual disability (congenital DM1) to the presence of subtle cognitive impairments [6,7].

Whilst the existence of cognitive impairment in DM1 is not in question, there is yet no consensus on the exact pattern of alteration. Some authors point to attentional, memory, and language deficits [3,8,9], suggesting a pattern of frontal lobe degeneration. Others have found results consistent with a pattern of visuospatial/constructive and executive function impairment [6,7,10,11].

In comparison with other neuromuscular diseases, patients with DM1 have a more serious functional disability and greater dependency in daily activities [12]. In addition, these patients show lower social engagement, more psychosocial problems, and poorer psychosocial well-being [13–16]. Likewise, in the DM1 population, particularly among congenital and childhood forms, comorbidity has been described with Autism Spectrum Disorder (ASD) [17], a disorder where social function is known to be compromised due to documented social cognition deficits.

Social cognition refers to a set of cognitive functions that enable a person to properly understand and interact in social situations. In other words, social cognition includes all processes implicated in the perception, interpretation, and generation of a response when facing the behaviours, interests, or intentions of others. It includes emotion perception, empathy, Theory of Mind (ToM) or mentalizing and attributional style. The theoretical framework of social cognition is still to be settled, but a trend exists towards distinguishing a more affective form of processing (ability to perceive and understand others’ emotional states) from another, more cognitive, form of processing (ToM and perspective-taking) [18]. In this regard, it is common to find studies assessing social cognition where a clear distinction is made, not only in conceptual terms but also based on neurobiological facts; emotion recognition (ventral stream) and the so-called Theory of Mind (dorsal pathway) [19]. ToM refers to the ability to perceive and understand other people’s behaviour, knowledge, intentions, emotions, and beliefs. A neuroimaging study has found damage to the dorsolateral frontal cortex and amygdala in DM1 [20], with other authors suggesting that in social cognition tasks in the general population, both structures are involved, along with other frontal structures [21].

In this regard, to date there have been relatively few studies addressing the role of social cognition in DM1, with most of this work having focused on facial emotion recognition [22–24]. Beyond the recognition of facial emotions, two studies have examined ToM in DM1 [20,25], both of which have led to the conclusion that ToM is affected in patients with DM1. Further, difficulties in facial memory ability associated with reduced visuo-constructive and visual memory ability have also been described in DM1 [26].

However, it is still unclear whether these difficulties are symptoms of the disease *per se* or are secondary to a more global general cognitive impairment [6]. In some conditions, such as intellectual disability, problems with social judgement, risk assessment, self-management, emotions or interpersonal relationships have been reported [27]. Though, social abilities in this group are associated with a more global impairment rather than being a specific characteristic of the illness. Indeed, a recent review points out that the high comorbidity found between DM1 and ASD might partly be explained by the underlying low IQ of patients in the sample [28], and therefore, the authors recommend to methodologically control this variable.

The aim of this study is to explore social cognition in DM1, taking into account certain cognitive mediator variables such as IQ, in order to clarify whether difficulties in this area are specific to DM1 or secondary to difficulties in other areas. Clarification of this issue will have important implications for the educational and social interventions directed towards improving the quality of life in this population.

## Method

This study has the approval of the Ethics Committee of the Donostia University Hospital, and written informed consent for participation and publication has been obtained from all participants.

### Participants

This study included a total of 38 volunteer patients with DM1 (19 women and 19 men) who were invited to participate in the study according to the consecutive order in which they attended the Neuromuscular Unit of the Neurology Service at Donostia Hospital. All patients had a molecular confirmation of the illness, and the patients’ clinical form was determined on the basis of the age of onset [29]. A control group of 38 subjects was recruited, paired in age (maximum allowed deviation ±5 years) and gender with the patient group. This group was composed of non-affected relatives of patients and healthy volunteers.

To address the objective of the study, inclusion criteria related to a normal IQ was set, in order to avoid the impact of an overall cognitive impairment on the outcome of social cognition tasks.

The inclusion criteria for both groups were (1) to be aged between 18 and 68 years (2) to have an IQ in the normal range (equal or over 85) at the time of assessment (3) the absence of any other neurological or psychiatric illness, and (4) abstinence from drugs or alcohol consumption.

### Materials

#### Neuromuscular assessment

1. Muscular Impairment Rating Scale [30], is a 5 point ordinal scale aimed at assessing the severity of distal and proximal muscular weakness in DM1. Patients’ data on this scale were retrieved from medical records.

#### Neuropsychological assessment

The neuropsychological assessment lasted approximately one hour. Some of the tests were used in an abbreviated form of the original test, in order to prevent the patients from the well-documented fatigue they suffer [31], which is known to have an impact on cognitive outcomes. Both a measure of overall cognitive functioning and a measure of attention performance were included:

1. Kaufman Brief Intelligence Test (K-BIT) [32]. This brief IQ estimation test was administered through its two scales: verbal (vocabulary subtest) and non-verbal (Matrices subtest) intelligence.
2. Digit span subtest from the Wechsler Adult Intelligence Scale (WAIS-III) [33]. This is a measure of immediate attention in which participants are requested to repeat sequences of numbers in both forward and backward recall conditions.

The tests below were used to assess three aspects of social cognition: emotion recognition using the Pictures of Facial Affect test, ToM using the Faux Pas test, and empathy using the Test of Cognitive and Affective Empathy.

1. Pictures of Facial Affect (POFA) [34]. This test was administered in order to assess the recognition of facial emotions. An abbreviated form of the original test, previously used in DM1 patients and composed of 28 pictures with a high rate of identification (80%) was employed in this study [24]. The stimuli used in this task included six men and seven women, expressing six basic emotions (happiness, sadness, fear, anger, disgust, surprise) and a neutral state, with each emotion being shown four times. All responses were elicited from the participants in a forced choice manner. Each correct response received 1 point. The psychometric properties of the POFA are largely assumed, since its construction was based on an extremely elaborate and highly reliable and valid system for determining and labelling the intensity of facial expressions of affect (FACS) [35].
2. The Faux Pas test [36]. This test assesses the ability of subjects to identify situations in which someone mistakenly says something they should not have (“*faux pas*”). Half of the 20 original stories were used; 5 faux pas and 5 control stories. Firstly, participants were requested to state whether a faux pas occurred or not. In the case of an affirmative answer, five open-ended questions were asked in order to assess subjects’ comprehension of the inappropriateness, intentions, and beliefs that a story character holds as well as his or her feelings. In the case of a negative answer, two open-ended control questions were asked. A correct response to each question received 1 point. Regarding the validity of the Faux-Pas Test, research has indicated that patients suffering mental illnesses or frontal lesions are typically impaired in their understanding of faux pas [37–40].
3. The Test of Cognitive and Affective Empathy (TECA) [41]. This is a 33-item self-report scale measuring both cognitive and affective aspects of empathy on a Likert-type five-point response format that ranged from 1 (totally disagree) to 5 (totally agree). The instrument assesses two affective dimensions of empathy (empathetic distress and empathetic joy, with 16 items) and two cognitive dimensions (perspective taking and emotional understanding, with 17 items). Scores on affective and cognitive empathy range from 16 to 80 and from 17 to 85 respectively. Regarding the validity of the TECA, it has shown strong convergent validity with high correlations with the Questionnaire Measure of Emotional Empathy (QMEE; [42]) and with the Spanish adaptation of the Interpersonal Reactivity Index (IRI; [43]).

### Statistical analysis

Firstly, group equivalence regarding age, years of study, and neuropsychological functioning was tested. Secondly, inter-group comparisons were conducted on social cognition measures. To do so, medians, interquartile ranges, Mann-Whitney *U* test and r effect size [44] were calculated when data distributional properties did not meet the assumptions for parametric statistical methods. When assumptions were met, means, standard deviations (SD), Student’s *t* test and Cohen’s d effect size [45] were calculated. We consider .20, .50, and .80 as small, medium, and large effect sizes when interpreting Cohen’s *d*, and .10, .30, .50 as small, moderate, and large effect sizes when interpreting *r*.

Correlation analyses were carried out for those social cognition measures with statistically significant differences between groups, in order to check for relations between those measures and CTG expansion size.

Additionally, correlations between age and social cognition measures were conducted separately in patients and controls, in order to look for any markers of aging related decline. Finally, correlations between IQ and social cognition measures were carried out for the whole sample, in order to analyse whether selected social cognition tests are sensitive to IQ in a sample with limited IQ range.

Correlations were calculated using Pearson’s *r* when variables met the normality assumption and Spearman’s rho when they did not.

## Results

No significant differences were found between patients and controls in terms of age, educational level, and cognitive functioning (IQ and attentional span) (Table 1).

**Table 1:** Socio-demographic and cognitive functioning data.

	Control Group (n = 38) Median (IQR)	Patient Group (n = 38) Median (IQR)	Mann Whitney *U*	*p*	*r*
**Socio-demographic data**					
Age	45.50 (37.75–54.00)	45.50 (37.75–53.25)	698.0	.803	0.029
Years of study	15.00 (11.75–17.00)	15.50 (11.00–18.00)	648.5	.442	0.088
**Cognitive functioning**					
IQ (K-BIT)	114.50 (104.00–124.00)	108.00 (101.00–117.25)	542.5	.062	0.214
Digit span (WAIS-III)	11.00 (9.00–15.00)	10.00 (7.00–15.00)	633.0	.353	0.106

IQR: interquartile range. Raw scores are presented except for IQ (standardized score X = 100; SD = 15)

The patients had a CTG expansion size ranging from 65 to 1667 and a mean score in MIRS of 2.27 (SD: 0.932). In our sample, 12 (31.6%) of the DM1 cases were juvenile onset, 21 (55.3%) were adult onset, and 5 (13.2%) were partial or late onset. Regarding inheritance, 86.8% inherited the illness from the father.

According to the differences on the social cognition tasks, significant differences were found only in the overall score of the POFA test with a moderate effect size (r = 0.31), with the controls obtaining a higher score than the patients (Tables 2 and 3).

**Table 2:** Differences in POFA and Faux Pas test between controls and patients.

	Control Group (n = 38) Median (IQR)	Patient Group (n = 38) Median (IQR)	Mann Whitney *U*	*p*	*r*
POFA	24.00 (23.00–26.00)	23.00 (22.00–24.00)	460.0**	.006	0.317
Faux Pas					
Control	38.00 (35.00–39.00)	37.50 (33.75–39.00)	712.5	.920	0.011
ToM	24.00 (19.00–31.50)	25.00 (21.00–30.50)	689.0	.731	0.039
Total	61.50 (56.00–67.00)	62.00 (56.00–68.25)	695.5	.783	0.032

IQR: interquartile range. Raw scores are presented.

**Statistically significant *p* < 0.01

**Table 3:** Differences in TECA between controls and patients.

	Control Group (n = 38) Mean (SD)	Patient Group (n = 38) Mean (SD)	Student *t*	*p*	*d*
TECA					
Perspective taking	53.26 (9.55)	50.71 (11.22)	1.068	.289	0.245
Emotional understanding	51.13 (12.30)	50.42 (9.25)	0.285	.777	0.065
Empathetic distress	51.92 (10.35)	50.24 (8.45)	0.777	.440	0.178
Empathetic joy	55.21 (10.55)	52.00 (11.39)	1.275	.206	0.292
Total	54.24 (9.91)	50.61 (8.95)	1.677	.098	0.384

SD: Standard deviation. Standardized scores are presented (X = 50; SD = 10).

Further analysis of the participants’ responses on the POFA revealed that patients showed a significantly lower score than the control group in the recognition of two emotions: anger and disgust (*U* = 439.0, *p* < .001, *r* = 0.39; *U* = 430.0, *p* < .001, *r* = 0.37, respectively). Table 4 shows frequencies and percentages of the number of correct answers given to each emotion by patients and controls. There we can see that 47.4% of healthy controls and 7.9% of patients correctly answered the four items of anger, whilst 63.2% of healthy controls and 31.6% of patients correctly answered the four items of disgust. An examination of the error pattern did not reveal a specific response pattern for these emotions in the DM1 group. In other words, anger and disgust were not systematically mistaken for another emotion. No correlation was found between the POFA total score and the number of CTG repeats (Spearman’s rho = .146, *p* = .380).

**Table 4:** Frequencies (f) and percentages (%) of correct answers for each emotion of the POFA test.

		Control group						Patient group		
		Number of correct answers						Number of correct answers		
Emotion	0 f(%)	1 f(%)	2 f(%)	3 f(%)	4 f(%)	0 f(%)	1 f(%)	2 f(%)	3 f(%)	4 f(%)
Happiness	0(0)	0(0)	0(0)	0(0)	38(100)	0(0)	0(0)	0(0)	0(0)	38(100)
Sadness	0(0)	2(5.3)	8(21.1)	15(39.5)	13(34.2)	0(0)	0(0)	7(18.4)	18(47.4)	13(34.2)
Fear	2(5.3)	2(5.3)	7(18.4)	7(18.4)	20(52.6)	2(5.3)	4(10.5)	4(10.5)	14(36.8)	14(36.8)
Anger	0(0)	2.6(1)	5.3(2)	44.7(17)	47.4(18)	0(0)	5.3(2)	7.9(3)	78.9(30)	7.9(3)
Disgust	0(0)	0(0)	10.5(4)	26.3(10)	63.2(24)	0(0)	5.3(2)	36.8(14)	26.3(10)	31.6(12)
Surprise	0(0)	0(0)	7.9(3)	23.7(9)	68.4(26)	0(0)	5.3(2)	13.2(5)	13.2(5)	68.4(26)
Neutral	0(0)	0(0)	2.6(1)	31.6(12)	65.8(25)	0(0)	0(0)	5.3(2)	28.9(11)	65.8(25)

Additionally, the results regarding the relationship between age and social cognition measures showed that there was a statistically significant negative correlation with POFA scores, but only in the patient group (Spearman’s rho = -.35; *p* = .03 in the patient group; Spearman’s rho = .15; *p* = .37 in the control group). No other outcome measure showed a statistically significant correlation with age, in either patients or in controls.

Finally, the results of the correlation between outcome measures and IQ revealed a statistically significant relationship between IQ and POFA scores (Spearman’s rho = .25; *p* = .028) and Faux Pas Test scores (Spearman’s rho = .26; *p* = .022), but not between IQ and TECA.

## Discussion

The results of this study, which aimed to analyse social cognition in patients with DM1, have partially ruled out the presence of specific difficulties in these abilities for this group of patients, when including only participants whose IQ is in the normal range. DM1 patients did not show specific difficulties in social cognition tasks which demand higher order cognitive processes (i.e. ToM tasks), but performed worse than controls on a task where a less cognitively demanding inference is required (facial emotion recognition).

It has been suggested that the difficulties shown by the DM1 population in social participation may be related to social cognition abilities, and several studies have emerged examining this topic in DM1. In particular, limitations in the ability to recognize facial emotions [22–24,46], as well as ToM and empathy deficits [20,25], have been reported. Nonetheless, these studies have some methodological limitations (small sample sizes, lack of a control group, or absence of an overall cognitive impairment measure) that could explain the differences with respect to the results obtained in our study, in which we have tried to overcome these limitations.

The patients in our study did not show poorer performance than the controls on either the Faux Pas Test or the TECA. To the best of our knowledge, only a few studies have employed other tools apart from facial emotion recognition in DM1. These studies have assessed social cognition through the Faux Pas test [22,25] and the Theory of Mind test (a modified Italian version of the Happé’s Strange Stories test) [20]. In the Faux Pas test, researchers found DM1 patients to be less sensitive to the emotional impact of a faux pas, whilst in contrast, patients failed on the more cognitive ToM test.

Similar to previous studies, our results confirm that DM1 patients have difficulties in facial emotion recognition tasks, specifically in anger and disgust, where patients’ scores are significantly lower than controls. This same result was reported by Kobayakawa and colleagues [22,46], using the Reading the Mind in the Eyes Test (RMET). Similarly, Takeda et al. [23] and Winblad et al. [24] reported lower scores in the DM1 group for anger and disgust, and additionally found differences in the recognition of fear.

Contrary to the findings of Winblad et al. [24], the failure to find a correlation between scores on the POFA test and CTG expansion size in our study could be taken to suggest that facial emotion recognition deficit is a specific deficit affecting DM1 patients as a whole group and is not dependent on genetic load. This idea is also supported by the results of Kobayakawa et al. [25] who found no correlation between CTG and RMET. With respect to the relationship between POFA scores and IQ, in our study we found this correlation to be statistically significant (see also Kobayakawa et al., who found a correlation between RMET and IQ [25]). Nonetheless, variations in IQ are unable to account for the significant differences in POFA scores that emerged between the patients and controls in our study. Thus, although sensitive to IQ level, facial emotion recognition impairment is not secondary to cognitive functioning, but instead appears to be inherent to the disease itself.

Taken together, our results—and most of those found in the reviewed literature—point towards a clear impairment in affectively loaded social cognition tasks (emotion recognition), whilst inconsistent results are found when using more cognitively loaded tasks (ToM). Moreover, the latter are usually story-based, which demands the functioning of other processes such as attention, text comprehension, and working memory—functions usually affected in patients with cognitive impairment. Taking into account that the DM1 population has, as a group, a lower IQ in comparison with the general population, one could hypothesize that the social difficulties identified in previous studies are secondary to their limitations at cognitive level [5,6,11]. The lack of results suggesting cognitive social cognition impairment in this study may possibly be due to the fact that the patients’ IQ was normal. This allowed us to avoid the bias that can be produced by general cognitive damage when performing such tasks. Indeed, the self-report was the only measure that did not correlate with IQ, suggesting a high cognitive load in the remaining assessment tools employed.

When looking for an aging effect, a negative correlation was found only between the POFA score and the age of the patients. In the last decade, longitudinal studies supporting the notion of an age-related decline in the DM1 population have emerged [47,48]. More specifically, an age-related decline of frontal and temporal functions has been described [3,8]. Facial emotion recognition impairment is a core and early developing feature in patients with fronto-temporal dementia [49,50]. Our finding that POFA scores are worse in older patients could reinforce the notion of a decline in fronto-temporal functions.

Nevertheless, this study also has certain limitations. First, although excluding low IQ patients from the study has allowed us to analyse social cognition whilst avoiding the bias that this implied, it has also reduced the representativeness of our sample (despite our sample being representative in terms of molecular damage). Additionally, the ecological validity of the tests used is arguable, given that these may not be representative of daily life situations where social cognition skills are required. However, this is a largely unavoidable issue when measuring social abilities with the tools currently available in such artificial contexts. A more natural way of studying the concept may be achieved by including reports or questionnaires from relatives who could provide more information about the adaptability and functionality of the patients being studied.

In summary, the results of this study suggest a more specific impairment of affective aspects of social cognition in DM1, while those social skills with greater cognitive load (at least ToM) may rely on intellectual processes. Therefore, in patients with cognitive deficit, impairment on these tasks could be secondary; a possibility that has already been put forward by other authors who have suggested a role for symptoms such as apathy, which is linked to overall cognitive status [14]. In this regard, our findings raise the question of whether the growing body of evidence on DM1 social cognition impairment should be regarded as a possible reflection of their overall cognitive difficulties.

In future work, it could be of interest to include other possible confounding or mediator variables such as those related to comorbid clinical symptomatology (apathy, depressive symptoms, etc.) or executive dysfunction, as suggested by a recent meta-analysis [51]. This would provide the scientific community with a greater understanding of the reasons why DM1 patients have such difficulties integrating family, work, and social life, and such information could consequently help towards raising their quality of life. Finally, our results suggest the possibility of opening a research discussion on this topic by considering facial emotion recognition as a possible marker of aging-related decline in DM1, as has been established in other types of dementia with which DM1 appears to share features.
